# Epidemiologic and genetic associations of female reproductive disorders with depression or dysthymia: a Mendelian randomization study

**DOI:** 10.1038/s41598-024-55993-8

**Published:** 2024-03-12

**Authors:** Shuyi Ling, Yuqing Dai, Ruoxin Weng, Yuan Li, Wenbo Wu, Ziqiong Zhou, Zhisheng Zhong, Yuehui Zheng

**Affiliations:** Reproductive Health Department, Shenzhen Traditional Chinese Medicine Hospital, The Fourth Clinical Medical College of Guangzhou University of Chinese Medicine, Shenzhen, 518000 Guangdong China

**Keywords:** Depression or dysthymia, Female reproductive disorders, Mendelian randomization, Causality, GWAS, Risk factors, Urogenital reproductive disorders

## Abstract

Observational studies have previously reported an association between depression and certain female reproductive disorders. However, the causal relationships between depression and different types of female reproductive disorders remain unclear in terms of direction and magnitude. We conducted a comprehensive investigation using a two-sample bi-directional Mendelian randomization analysis, incorporating publicly available GWAS summary statistics. Our aim was to establish a causal relationship between genetically predicted depression and the risk of various female reproductive pathological conditions, such as ovarian dysfunction, polycystic ovary syndrome(PCOS), ovarian cysts, abnormal uterine and vaginal bleeding(AUB), endometriosis, leiomyoma of the uterus, female infertility, spontaneous abortion, eclampsia, pregnancy hypertension, gestational diabetes, excessive vomiting in pregnancy, cervical cancer, and uterine/endometrial cancer. We analyzed a substantial sample size, ranging from 111,831 to 210,870 individuals, and employed robust statistical methods, including inverse variance weighted, MR-Egger, weighted median, and MR-PRESSO, to estimate causal effects. Sensitivity analyses, such as Cochran's Q test, MR-Egger intercept test, MR-PRESSO, leave-one-out analysis, and funnel plots, were also conducted to ensure the validity of our results. Furthermore, risk factor analyses were performed to investigate potential mediators associated with these observed relationships. Our results demonstrated that genetic predisposition to depression or dysthymia was associated with an increased risk of developing PCOS (OR = 1.43, 95% CI 1.28–1.59; P = 6.66 × 10^–11^), ovarian cysts (OR = 1.36, 95% CI 1.20–1.55; P = 1.57 × 10^–6^), AUB (OR = 1.41, 95% CI 1.20–1.66; P = 3.01 × 10^–5^), and endometriosis (OR = 1.43, 95% CI 1.27–1.70; P = 2.21 × 10^–7^) after Bonferroni correction, but no evidence for reverse causality. Our study did not find any evidence supporting a causal or reverse causal relationship between depression/dysthymia and other types of female reproductive disorders. In summary, our study provides evidence for a causal relationship between genetically predicted depression and specific types of female reproductive disorders. Our findings emphasize the importance of depression management in the prevention and treatment of female reproductive disorders, notably including PCOS, ovarian cysts, AUB, and endometriosis.

## Introduction

Depression stands as the most prevalent psychiatric disorder worldwide. In 2017, the World Health Organization (WHO) reported that over 300 million individuals, accounting for 4.4% of the global population, suffered from depression^1^. From 1990 to 2017, the global incidence of depression has increased 49.86%^2^. Moreover, it is projected by WHO that depression will emerge as a principal contributor to the global burden of disease by 2030^3^.

Depression has been found to have associations with various female reproductive disorders. Its prevalence has been estimated to be approximately 31% in patients with PCOS^4^, ranging from 11% ^5^ to 27% ^6^ and 31.3% ^7^ in females with infertility, 15.6% in those with AUB^8^, 18.6% in individuals with spontaneous abortion^9^, and 27% in patients with ovarian cancer^10^. Moreover, it is noteworthy that depression presents a substantial risk element for the onset of gestational diabetes among expectant mothers, exhibiting a correlated augmented risk of 29%^11^. Additionally, patients diagnosed with PCOS exhibit 4 times greater likelihood of developing depression in comparison to women without PCOS^12^. Furthermore, it is imperative to acknowledge that women have a higher prevalence of depression compared to men, with a risk ratio of approximately 2:1^13^. This significant difference emphasizes the importance of considering the impact of depression on women's reproductive health. Previous studies primarily relied on observational studies, including case–control studies^14,15^ and cross-sectional studies^7,16^ and cohort studies^9,17^. Although these studies provided an estimate of the relationship between depression and reproductive status, the causal relationship remains unclear.

The traditional design of observational studies comes with inherent limitations, which often challenge the inference of causality. Potential mixed bias and reverse causality may lead to biased correlations and conclusions^18^. Furthermore, conducting randomized controlled trials (RCTs), recognized as the gold standard for establishing causal inference, is unethical and impractical in this case due to the need for substantial human resources, time-consuming follow-up, and the inability to randomly assign depression to different individual groups. To overcome these limitations, Mendelian randomization (MR) has been increasingly employed to infer credible causal relationships between risk factors and disease outcomes^19^. MR utilizes genetic variation, randomly distributed during meiosis, as an instrumental variable associated with environmental exposure. This approach enables the evaluation of the causal association between depression/dysthymia and different types of female reproductive disorders^20^. Two-sample bi-directional MR analysis explores both directions of causality, providing a comprehensive comprehension of the association between exposure and outcome variables. MR studies have been conducted to explore the causal relationship between depression and PCOS^21^, endometriosis^22^, and ovarian cancer^23^. However, to date, there is a lack of systematic MR studies that have revealed the causal association between depression/dysthymia and other female reproductive disorders.

In this study, we conducted a two-sample bi-directional MR analysis using publicly available genome-wide association study (GWAS) summary statistics. Our study represents the first comprehensive report of the causal relationships between depression/dysthymia and 14 common female reproductive disorders, including ovarian dysfunction, PCOS, ovarian cysts, AUB, endometriosis, leiomyoma of the uterus, female infertility, spontaneous abortion, eclampsia, pregnancy hypertension, gestational diabetes, excessive vomiting in pregnancy, cervical cancer and uterine/endometrial cancer, through the application of MR analysis. The findings of this investigation hold the potential to yield significant insights into the causal links between depression/dysthymia and female reproductive disorders, consequently offering constructive recommendations for the implementation of preventive intervention strategies.

## Methods

### Study design

This study utilized a two-sample bi-directional MR analysis to examine the causal effect of depression or dysthymia on female reproductive disorders, leveraging by GWAS summary statistics. We also employed instrumental-variable analysis, which emulates a RCT by simulating the random allocation of single nucleotide polymorphisms (SNPs) in offsprings.

To ensure the robustness of our MR design, we adhered to the guidelines outlined in STROBE-MR^24^ and carefully evaluated three crucial assumptions. First, the genetic instrument used should strongly predict the exposure of interest, as determined by meeting the genome-wide significance threshold (P < 5 × 10^–8^) for the instrumental variants^25^. Second, the genetic instruments must be independent of any confounding factors that might influence both the exposure and the outcome of interest^26^. At last, it is crucial to establish that the genetic instruments solely impact the outcome through their association with the exposure, rather than through alternative pathways^27^.

In the reverse MR analysis, we employed a relaxed P threshold (P < 5 × 10^–6^) for the instrument-exposure association in order to include more SNPs for traits with limited SNPs (≤ 3) after linkage disequilibrium (LD) pruning. This approach has been used in many previous MR studies^28–30^. However, it may increase the risk of violating the first assumption of MR design.

### Data sources: exposure and outcome variables in GWAS

The FinnGen consortium (https://www.finngen.fi/fi, accessed on July 10, 2023) provided GWAS data for exposure (depression or dysthymia: ICD-10 code F3[2, 3]/F341, 48,847 cases & 225,483 controls) and outcomes: ovarian dysfunction (ICD-10 code E28, 2,010 cases & 200,581 controls), PCOS (ICD-10 code E282, 13,142 cases & 107,564 controls), ovarian cysts (ICD-10 code N83[0–2], 20,750 cases & 107,564 controls); uterine conditions: AUB (ICD-10 code N93, 10,319 cases & 107,564 controls), endometriosis(ICD-10 code N80, 15,088 cases & 10,7564 controls), leiomyoma of uterus(ICD-10 code D25, 31,661 cases & 179,209 controls); fertility or pregnancy-related diseases: female infertility (ICD-10 code N97, 13,142 cases & 107,564 controls), spontaneous abortion (ICD-10 code O03, 16,906 cases & 149,622 controls), eclampsia (ICD-10 code O15, 452 cases & 194,266 controls), pregnancy hypertension (ICD-10 code O10|O11|O13|O14|O15|O16, 14,727 cases & 196,143 controls), gestational diabetes (ICD-10 code O244, 13,039 cases & 197,831 controls), excessive vomiting in pregnancy (ICD-10 code O21, 2,361 cases & 179,899 controls). The GWAS data from the UK Biobank study (http://www.nealelab.is/uk-biobank/) provided additional outcomes, including cervical cancer (1450 cases & 192,703 controls) and uterine/endometrial cancer (906 cases & 193,247 controls). Detailed information about the characteristics of the studies and consortia used can be found in Additional file 1: Table S5.

As per the International Statistical Classification of Diseases and Related Health Problems 10th Revision, depression or dysthymia is a multifaceted mental health disorder encompassing various conditions such as depressive episode, recurrent depressive disorder, and dysthymia. Depressive episode is characterized by symptoms such as low mood, reduced energy, decreased activity, loss of interest, and difficulty concentrating. The severity of the symptoms can range from mild to moderate or severe, depending on their number and intensity. Recurrent depressive disorder involves repeated episodes of depression without any history of mania, and the severity and duration can vary. Dysthymia, on the other hand, is a chronic form of depression that persists for several years but does not meet the criteria for recurrent depressive disorder.

### MR analysis

To identify the causal relationship between depression/dysthymia and female reproductive disorders, three different MR methods, namely random effect inverse variance weighting (IVW), MR-Egger, weighted median (WM), and MR-PRESSO were utilized to address heterogeneity of variation and pleiotropic effects. Using multiple estimators in MR analysis improves the robustness and consistency of our findings by accounting for potential biases and uncertainties. Each estimator has unique strengths and limitations and makes different assumptions about genetic instrument validity and pleiotropy, which could affect the accuracy of estimates. By utilizing multiple estimators, we can evaluate the sensitivity of our results to different assumptions and increase confidence in the validity of our findings while mitigating concerns related to underlying assumptions. SNPs and abnormal values associated with female reproductive status, as identified by MR-PRESSO, were excluded^31^. IVW served as the primary outcome, while MR-Egger and weighted median were employed to improve the estimation of IVW, as they offer more reliable estimates in a broader range of scenarios, albeit with lower efficiency (wider confidence intervals). MR-Egger, although allowing for pleiotropic effects in all genetic variations, assumes that such effects are independent of the association between variation and exposure^32^. The weighted median method permits the inclusion of invalid instruments under the assumption that at least half of the instruments used in MR analysis are valid^33^. In IVW analysis, the weighted regression slope of the SNP result, showing effect on the SNP exposure with the intercept constrained to zero, represents the estimated outcome. For significant estimates, the MR-Egger intercept test and leave-one-out analysis were employed to further assess horizontal pleiotropy. Cochran's Q test was also used to identify heterogeneity. A funnel plot was utilized to evaluate possible directional pleiotropy, akin to assessing publication bias in meta-analysis.

Furthermore, prior to MR analysis, stringent filtering steps were implemented to ensure SNP quality. Firstly, linkage disequilibrium (LD, R^2^ ≥ 0.001 within 10 MB) was aggregated. Secondly, SNPs were required to reach the genome-wide significance threshold of P < 5 × 10^–8^ in relation to the relevant exposure. Thirdly, we assessed the strength of our instrument variables using two parameters: the proportion of variance explained (R^2^) and the F statistic. The R^2^ was calculated as R^2^ = β^2^ × 2 × MAF × (1 − MAF), where β represents the estimated effect and MAF indicates the minor allele frequency^34^. The F statistic was calculated using the formula F = [(N – k − 1)/k] × R^2^/(1 − R^2^), where N represents the sample size, k represents the number of included SNPs, and R^2^ represents the proportion of variance explained by the genetic variants^34^. The obtained F statistic values ranged from 215 to 400, as outlined in Additional file 1: Table S1, strongly indicating that the selected genetic variants effectively serve as suitable proxies for the investigated exposure^35,36^.

### Risk factors

In order to investigate the genetic mechanisms that link depression/dysthymia with female reproductive disorders, we conducted MR analyses using depression/dysthymia as exposure and several potential mediators as outcomes. These potential mediators included drinking, smoking, coffee intake, body mass index (BMI), circulating leptin levels, obesity, fasting insulin, insulin secretion rate, and diabetes^37–39^. GWAS summary data for these potential mediators were obtained from the IEU OpenGWAS database (https://gwas.mrcieu.ac.uk/, accessed on August 2, 2023)^40^. Detailed information regarding each data source can be found in Table 1. Depression/ dysthymia were considered as exposures, while the aforementioned potential risk factors were treated as outcomes for Mendelian randomization analysis. The primary results were evaluated based on estimates derived from IVW. Statistical significance was defined as P < 0.05.

**Table 1:** Data source for risk factors related to female reproductive disorders.

Traits	Category	Consortium	Sample size	Ancestry	GWAS ID
Drinking	Binary	UK Biobank	360,726	European	ukb-d-20117_2
Smoking	Binary	UK Biobank	91,353	European	ukb-d-22506_111
Coffee intake	Categorical ordered	UK Biobank	428,860	European	ukb-b-5237
BMI	Continuous	UK Biobank	461,460	European	ukb-b-19953
Circulating leptin levels	Continuous	EBI	21,758	European	ebi-a-GCST90012076
Obesity	Binary	UK Biobank	463,010	European	ukb-b-15541
Fasting insulin	Continuous	EBI	16,386	Hispanic or Latin American	ebi-a-GCST90002239
Insulin secretion rate	Continuous	EBI	527	European	ebi-a-GCST004488
Diabetes	Binary	UK Biobank	461,578	East Asian	ukb-b-10753

### Statistical analysis

The statistical analyses were conducted using the TwoSampleMR package (version 0.5.7) and MRPRESSO package (version 1.0) within the R environment (version 4.3.0).To address the issue of multiple testing, a Bonferroni correction was applied by setting the significance threshold at 0.05 divided by the number of MR estimates (14), resulting in a Bonferroni-corrected P-value of 3.57 × 10^−3^. Additionally, associations with a P-value less than 0.05 but not yet meeting the Bonferroni-corrected threshold were considered nominally significant^41^.

### Ethics approval and consent to participate

The data utilized in this study were obtained from publicly available, de-identified sources and were originally collected from participant studies that had already received approval from an ethics committee regarding human experimentation. As a result, no additional ethical approval was necessary for this particular study.

## Results

### MR analysis

**Figure 1:**
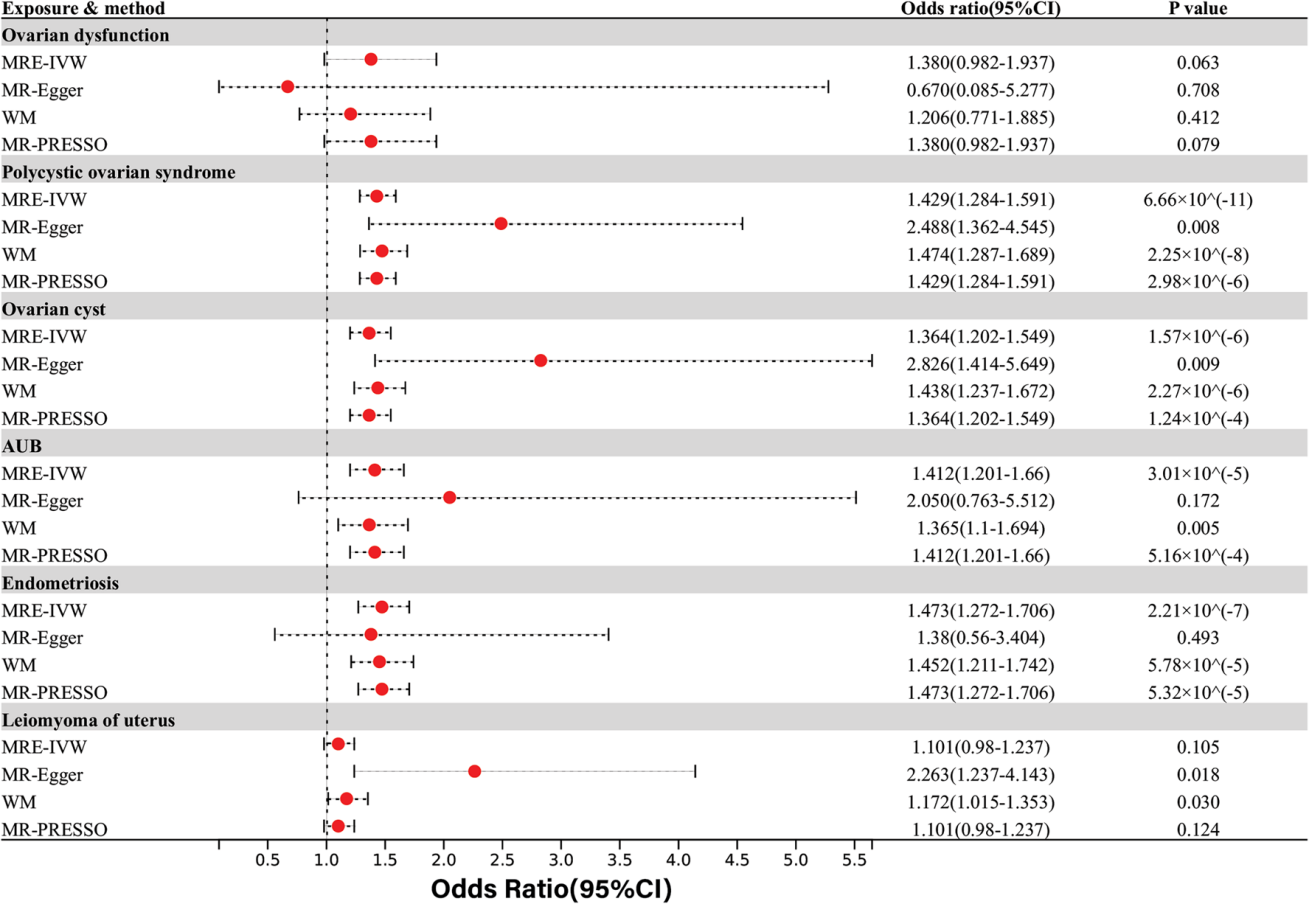
Causal effects for depression or dysthymia on ovarian and uterine-related disorders. Summary of the Mendelian randomization (MR) estimates derived from the inverse-variance weighted (IVW), MR-Egger, weighted median (WM) and MR-PRESSO methods.

Within the spectrum of gynecological conditions encompassing ovarian or uterine disorders, the IVW analysis revealed a significant positive association between depression/dysthymia and several conditions, including PCOS (OR = 1.43, 95% CI 1.28–1.59; P = 6.66 × 10^–11^), ovarian cysts (OR = 1.36, 95% CI 1.20–1.55; P = 1.57 × 10^–6^), AUB (OR = 1.41, 95% CI 1.20–1.66; P = 3.01 × 10^–5^), and endometriosis (OR = 1.47, 95% CI 1.27–1.71; P = 2.21 × 10^–7^). These findings were consistent with other MR method results. Additionally, the MR-Egger and WM analyses suggested a nominal correlation between depression/dysthymia and leiomyoma of the uterus. The IVW and MR-PRESSO analyses also showed consistent directions, but without statistical significance. However, there was no observed causal relationship between depression/dysthymia and ovarian dysfunction (OR = 1.38, 95% CI 0.98–1.94; P = 0.063) (Fig. 1).

**Figure 2:**
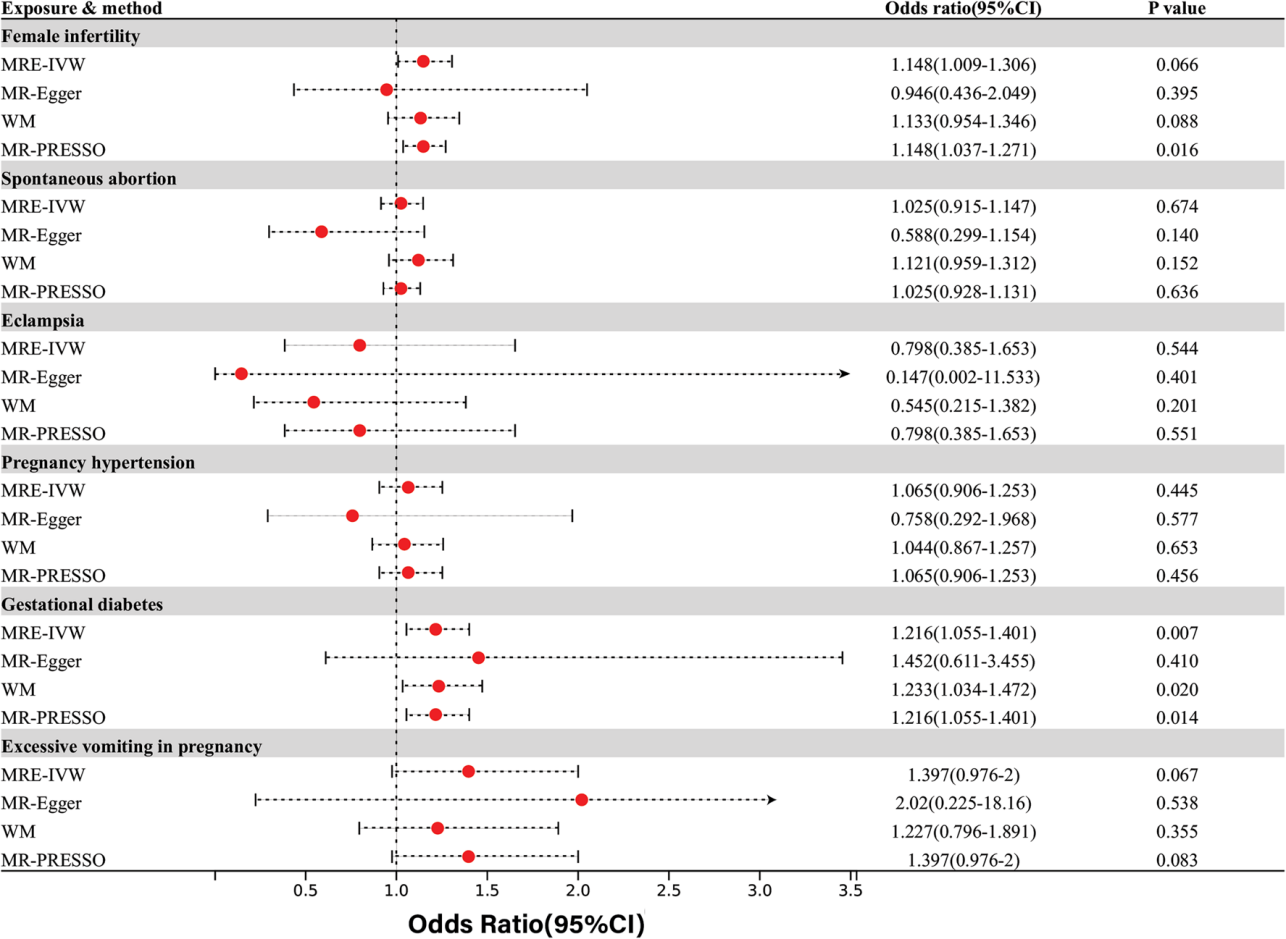
Causal effects for depression or dysthymia on fertility and pregnancy-related disorders.

Among fertility and pregnancy-related diseases, evidence suggested a nominal correlation between depression/dysthymia and the risk of gestational diabetes through IVW analyses (OR = 1.22, 95% CI 1.06–1.40; P = 0.007). This association has been consistently observed in other MR analyses, except for the MR-EGGER analysis. Furthermore, MR-PRESSO analysis indicated a nominally significant correlation between depression/dysthymia and female infertility (OR = 1.15, 95% CI 1.04–1.27; P = 0.016). However, this association was not found to be statistically significant in other MR analysis methods, which showed inconsistent results. In addition, multiple analyses showed that there was no statistically significant association between depression/dysthymia and other pregnancy-related conditions such as spontaneous abortion, eclampsia, pregnancy-induced hypertension, and hyperemesis gravidarum(Fig. 2).

**Figure 3:**
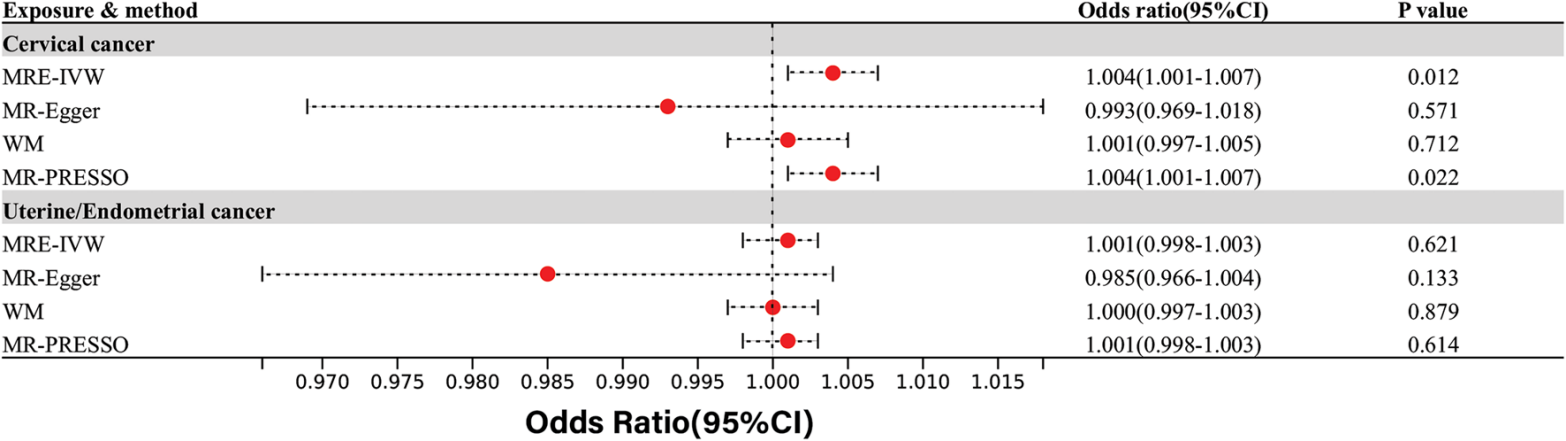
Causal effects for depression or dysthymia on reproductive-related cancers.

In the context of common reproductive-related cancers, IVW and MR-PRESSO analyses revealed a nominally significant correlation between depression/dysthymia and cervical cancer, while MR-Egger analysis showed the opposite direction without statistical significance. Furthermore, there was no observed causal relationship between depression/dysthymia and uterine/endometrial cancer(Fig. 3).

Our analysis of reverse causality, specifically focusing on depression as the outcome and female reproductive status as the exposure, yielded no evidence in support of reverse causality. Among all the factors examined, PCOS showed nominal statistical significance in both the IVW analysis and MR PRESSO analysis(see Additional file 1: Table S3). However, it is important to interpret these findings cautiously as they do not provide definitive evidence for a causal relationship.

### Sensitivity analysis

**Figure 4:**
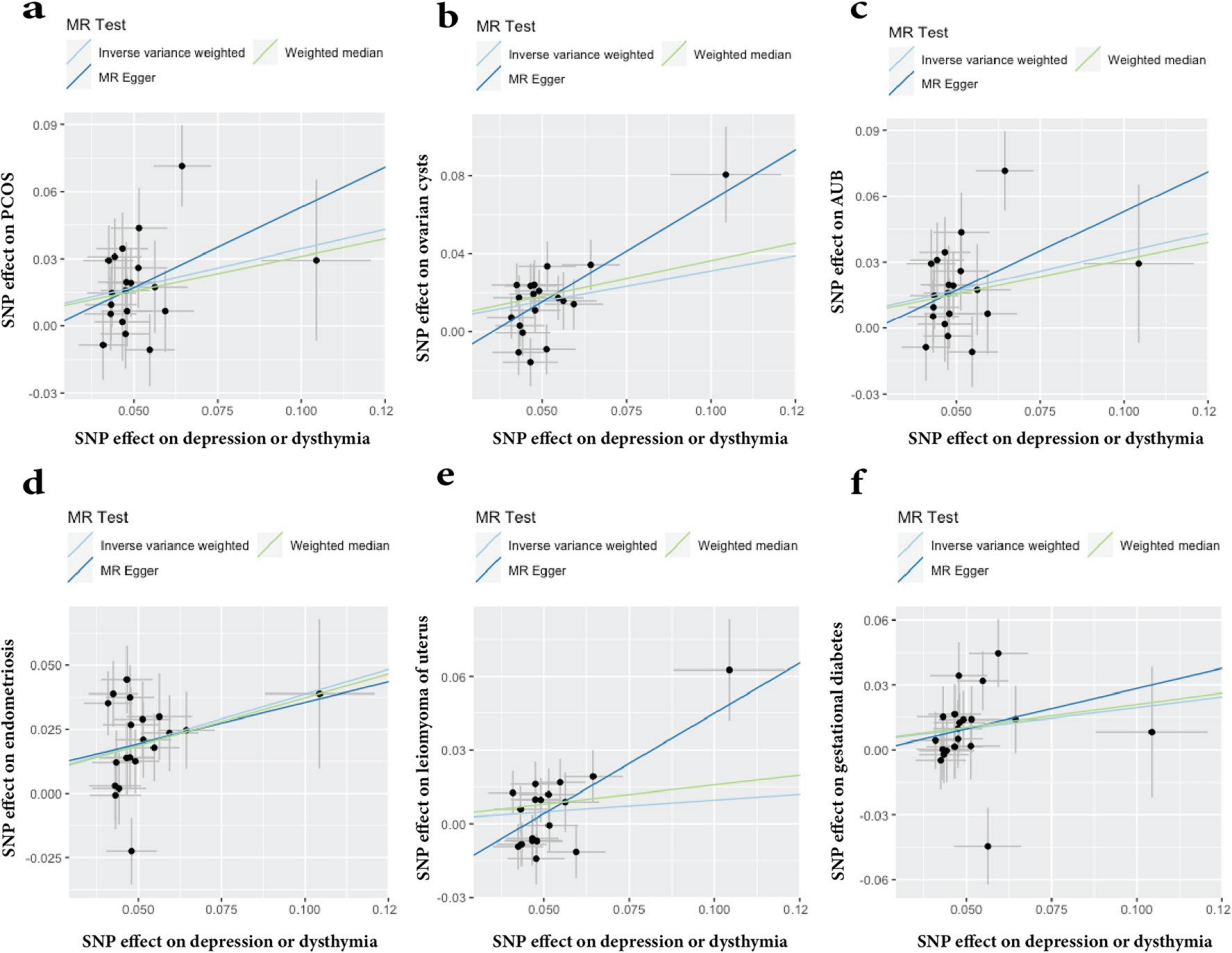
Scatter plots depicting the impact of genetically predicted depression/dysthymia on the risk of female reproductive disorders.

**Figure 5:**
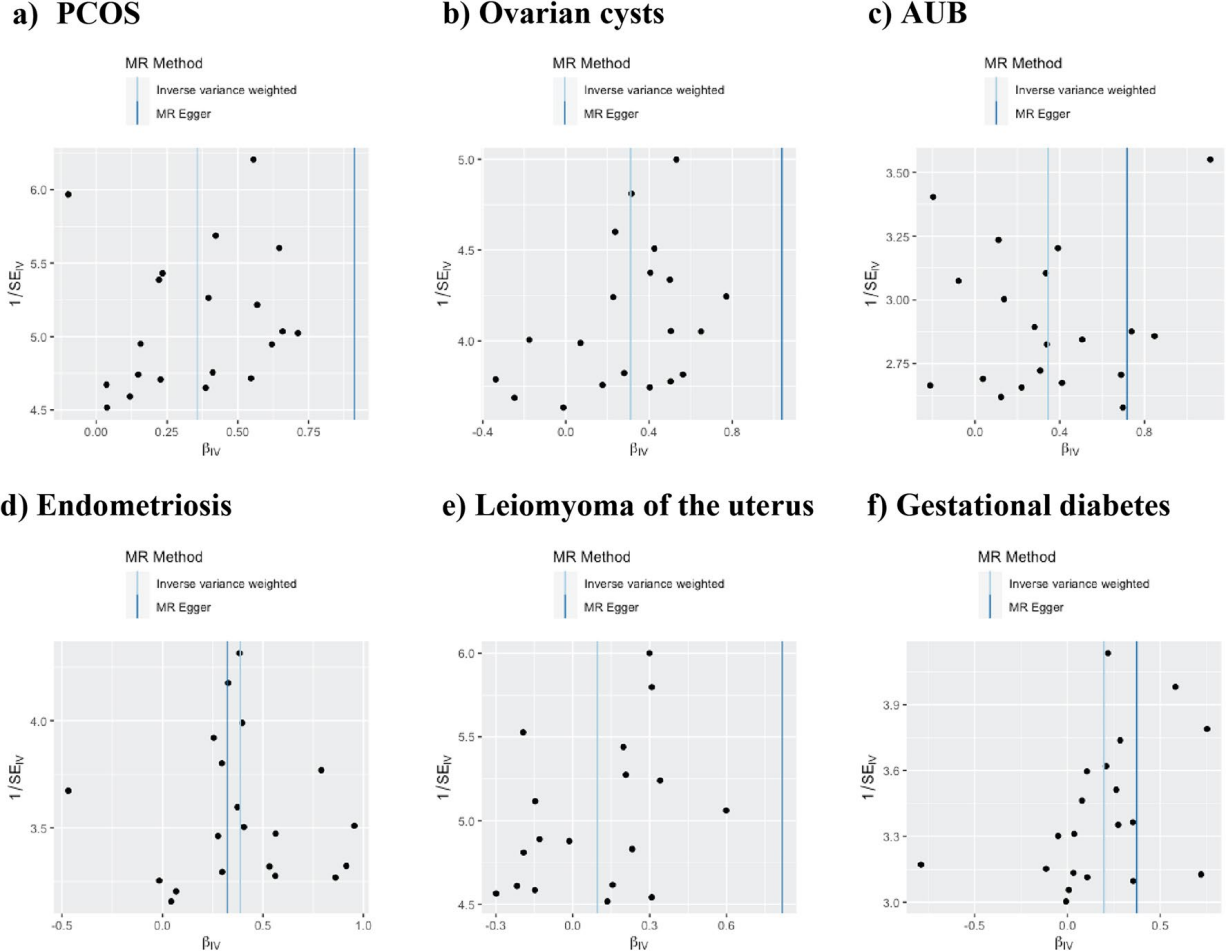
Funnel plots depicting the impact of genetically predicted depression or dysthymia on the risk of female reproductive disorders. The funnel plots show the Inverse variance weighted and MR-Egger MR estimate of each depression/dysthymia single-nucleotide polymorphism with different female reproductive disorders versus 1/standard error (1/SE_IV_). (**a**) PCOS; (**b**) ovarian cysts; (**c**) AUB; (**d**) endometriosis; (**e**) leiomyoma of the uterus; (**f**) gestational diabetes.

To evaluate the robustness of the aforementioned findings, a series of sensitivity analyses were conducted, including Cochran's Q test, MR-Egger intercept test, and MR-PRESSO global test (Table 2). Heterogeneity was observed in the Q test analysis between depression/dysthymia and pregnancy hypertension (Q = 27.97, P = 0.045), while other outcomes did not exhibit heterogeneity. The use of random-effects IVW as the main estimation method adequately accounted for acceptable heterogeneity^42^. Additionally, excepting P value of leiomyoma of uterus (MR-Egger Intercept = -0.04, P = 0.031), P values of the MR-Egger intercept tests from other outcomes were above 0.05, indicating the absence of pleiotropic bias in the examined female reproductive disorders, except for leiomyoma of uterus(Fig. 4). Furthermore, leave-one-out analysis revealed that no SNP significantly influenced the results, and funnel plots displayed symmetrical distributions (Fig. 5; Additional file 2: Figs. S2 and S3), signifying the absence of estimation violations. No heterogeneity was detected in the other analyses. The sensitivity analysis results of the reverse causality analysis are presented in the Additional file 1: Table S4.

**Table 2:** Sensitivity analysis of the causal association between depression/dysthymia and the risk of female reproductive disorders.

Outcome	Cochran Q value	Q test P	MR-Egger intercept	P	MR-PRESSO P value
Ovarian dysfunction	22.437	0.263	0.036	0.495	0.290
PCOS	29.854	0.054	−0.028	0.095	0.091
Ovarian cysts	27.109	0.102	−0.037	0.134	0.134
AUB	22.141	0.277	−0.019	0.463	0.292
Endometriosis	27.213	0.100	0.003	0.133	0.142
Leiomyoma of uterus	27.331	0.053	−0.037	0.031	0.059
Female infertility	11.865	0.891	0.010	0.624	0.894
Spontaneous abortion	14.608	0.747	0.028	0.119	0.744
Eclampsia	23.613	0.211	0.085	0.451	0.214
Pregnancy hypertension	27.966	0.045	0.017	0.488	0.044
Gestational diabetes	23.247	0.227	−0.009	0.688	0.232
Cervical cancer	18.231	0.441	0.001	0.395	0.461
Uterine/endometrial cancer	16.717	0.543	0.001	0.118	0.549

### Risk factors analysis

To investigate the potential factors that mediate the association between depression/dysthymia and an increased risk of female reproductive pathological conditions, we utilized MR methods to evaluate its impact on several common risk factors associated with these conditions. The results presented in Table 3 demonstrate that the causal effect of depression or dysthymia on female reproductive disorders remained unaffected by the potential risk factors examined, except for drinking.

**Table 3:** Risk factors analysis.

Outcomes	IVW Causal effect (95% CI)	P value	Heterogeneity Q value	P value	MR-Egger Intercept	P value
Drinking	1.016(1.007–1.025)	0.001	41.833	0.001	−0.001	0.672
Smoking	0.999 (0.991–1.008)	0.885	22.509	0.210	−0.002	0.358
Coffee intake	1.024 (0.992–1.057)	0.141	57.301	2.91 × 10^−6^	0.009	0.202
BMI	0.923 (0.848–1.004)	0.061	214.664	2.19 × 10^−37^	0.003	0.872
Circulating leptin levels	0.927 (0.811–1.061)	0.272	19.599	0.188	−0.033	0.197
Obesity	0.999 (0.996–1.003)	0.687	36.406	0.003	4.56 × 10^−6^	0.995
Fasting insulin	0.99 (0.922–1.062)	0.771	18.934	0.396	−0.003	0.820
Insulin secretion rate	0.67 (0.433–1.038)	0.073	13.667	0.691	−0.027	0.776
Diabetes	0.994 (0.983–1.005)	0.273	52.285	1.20 × 10^−6^	−0.001	0.629

## Discussion

Based on large-scale GWAS data from the Finngen and UK Biobank, this study employed a variety of MR approaches to comprehensively examine the potential causal or reverse association between depression/dysthymia and female reproductive disorders. Our research provides compelling evidence that individuals with depression /dysthymia have a significantly higher risk of developing the following conditions: PCOS (42.9% increased risk), ovarian cysts (36.4% increased risk), AUB (41.2% increased risk), and endometriosis (47.3% increased risk). These results call for more attentions on depression/ dysthymia management and treatment in term of reducing female reproductive diseases such as endometriosis, PCOS and AUB. For instance, integrating screening for depressive symptoms during routine gynecological exams, implementing non-pharmacological interventions such as cognitive-behavioral therapy and exercise, ensuring careful administration of pharmacological treatments under professional guidance, and strengthening social support systems are all crucial components in addressing this issue.

The utilization of MR in our investigations provides a decreased susceptibility to biases stemming from confounding factors or reverse causation, as compared to observational epidemiological studies. In general, the statistical power of the IVW approach is significantly higher compared to other MR approaches, particularly MR-Egger^43^. Confidence intervals were derived from the same statistical equations used to calculate P values. Consequently, it is expected that the MR-Egger results, with lower statistical power, would yielded wider confidence intervals and non-significant P values when compared to IVW in the present study. Thus, IVW was primarily employed as the main method for identifying potentially significant findings. Sensitivity analyses and other MR methods were utilized to ensure the robustness of the IVW estimates. If there is any evidence of horizontal pleiotropy, IVW estimates could be biased. In such cases, MR-Egger estimates should be considered because this method accommodates the unbalanced or directional effects of horizontal pleiotropy across all SNPs^44^. Most MR analyses also require consistent beta directions across all MR approaches^37,45^, as is the case in our study, which means that the beta coefficients of all MR approaches should consistently be positive or negative to obtain a robust conclusion. While it is essential to exercise caution when interpreting causal associations derived from MR due to the presence of untestable assumptions inherent to this method, the convergence of our estimates across various methodologies and analytical approaches strongly supports the causal involvement of depression or dysthymia in the etiology of female reproductive disorders.

Our study provides initial evidence indicating that genetically predicted depression/dysthymia may be a causal factor, rather than a consequence, of various female reproductive diseases. Specifically, our results demonstrate significant associations between genetically predicted depression/dysthymia and the following conditions: Endometriosis (OR = 1.47, 95% CI 1.27–1.71), PCOS (OR = 1.43, 95% CI 1.28–1.59), AUB (OR = 1.41, 95% CI 1.20–1.66), and Ovarian cysts (OR = 1.36, 95% CI 1.20–1.55). These findings align with other MR studies that have also suggested depression as a risk factor for PCOS^21^ and endometriosis ^46^.

It is important to emphasize that our study did not establish a causal relationship between depression and some other conditions, including ovarian dysfunction, leiomyoma of uterus, female infertility, spontaneous abortion, eclampsia, pregnancy hypertension, excessive vomiting in pregnancy, cervical cancer, or uterine/endometrial cancer. Additionally, our analysis of reverse causality found no evidence supporting such a reverse link. Previous MR studies have also assessed the causal relationship between depression and ovarian cancer, revealing no significant association^23^. Moreover, a cohort study supports our findings by indicating that depression is unlikely to be the cause of excessive vomiting in pregnancy^47^. However, it should be noted that several observational studies suggest a higher likelihood of depression among patients with ovarian dysfunction^48^, female infertility^49^, leiomyoma of uterus^50^, abortion^51^, pregnancy hypertension^52^, eclampsia^53^, cervical cancer^54^ and endometrial cancer^55^. These studies propose that depression may either result from these conditions or contribute to their development. The discrepancies observed between the results of MR studies and observational studies, as well as the controversies within the latter, can be attributed to confounding factors and biases inherent in real-world epidemiological studies. Notably, MR, which functions as an analogous approach to randomized controlled trials, emerges as a more effective tool for drawing causal inferences due to its reduced susceptibility to confounding influences^23^.

As indicated by the risk factor analyses, drinking behaviors may play a role in the susceptibility of female reproductive disorders linked to depression or dysthymia. Research has demonstrated a positive association between alcohol dependence and depression, indicating the potential involvement of interconnected neurobiological mechanisms^56,57^. It is widely recognized that alcohol negatively affects female reproduction^38^. However, whether depression influences women's reproductive health through similar neural mechanisms remains uncertain.

Numerous mechanisms have been proposed to elucidate the impact of depression on female reproductive status. It is widely believed that depression exerts its influence on female reproduction through the hypothalamic–pituitary–adrenal (HPA) axis and the hypothalamic-pituitary-ovarian (HPO) axis^58–61^.Corticotropin-releasing hormone (CRH), originating from the hypothalamus, is implicated in various reproductive processes, including follicular development, ovulation, and luteolysis in the ovarian CRH^62,63^. Furthermore, recent findings indicate that CRH-R1 is expressed in reproductive tissues such as the ovary, endometrium, and myometrium, and plays a pivotal role in regulating reproductive functions^62–64^.In addition, abnormal lactic acid metabolism and glycolysis may serve as a link between depression and reproductive diseases. Studies conducted on mice have demonstrated that the modulation of lactic acid homeostasis can influence neuronal excitability and depression-like behavior^65^. Associations have been found between lactic acid and uterine remodeling^66^, abnormal glycolysis and ovarian cancer^67^, as well as oocyte quality of PCOS patients^68^.Moreover, depression often coexists with an imbalance in intestinal flora^69–71^. Disruptions in gastrointestinal ecology actively contribute to the development and metastasis of gynecological tumors, such as cervical cancer, endometrial cancer, and ovarian cancer^72^. Notably, several studies have revealed that the use of probiotics can ameliorate depressive symptoms and regulate sex hormone levels, offering potential therapeutic benefits for women with PCOS and gestational diabetes^73–75^.Besides, chronic inflammation and oxidative stress are prominent features of depression^76,77^ and pathological conditions pertaining to female reproductive health, including PCOS, ovarian dysfunction, endometriosis, gestational diabetes, and leiomyoma of uterus^78–82^. Inflammatory processes are intertwined with the onset of depression, which can further exacerbate the inflammatory response and detrimentally impact the reproductive system^83–85^.

However, it is essential to acknowledge that our study possesses several inherent limitations that necessitate cautious interpretation. Firstly, the generalizability of our findings to diverse ethnic groups with distinct lifestyles and cultural backgrounds may be limited, as our study exclusively focused on individuals of European ancestry. Secondly, it is essential to recognize the inherent challenges of MR analyses, which rely on the random allocation of genetic variants, in fully disentangling mediation from pleiotropy. Plausible scenarios exist wherein genetic variants within our genome may exert simultaneous influences on multiple phenotypes. Furthermore, the absence of significant findings in our study can be attributed to statistical power constraints and inadequate representation of SNPs. The persisting issue of "missing heritability" in various polygenic diseases and traits, particularly psychiatric disorders, may be addressed through ongoing research utilizing SRS and LRS technologies to explore rare variants^86^. Consequently, our ability to draw definitive conclusions regarding true causal relationships is impeded. Although we attempted to enhance sensitivity by relaxing the exposure threshold in our reverse causality MR analysis, the limited number of strongly associated SNPs analyzed may result in reduced statistical power to detect significant associations or limit the generalizability of the findings. Given the inherent limitations of the Finngen and UK Biobank datasets, such as our inability to access participants' individual data, it is imperative that future studies are conducted to validate causal relationships and explore underlying mechanisms. Such investigations are crucial for generating meaningful clinical recommendations that accurately inform medical practice.

In conclusion, utilizing extensive genetic summary data, our study provides strengthened evidence supporting a causal link between depression/dysthymia and the risk of specific female reproductive disorders, including endometriosis, PCOS, AUB, ovarian cysts and gestational diabetes. However, the reverse causal relationship between these conditions and depression remains undetermined. These findings highlight the significance of mental health in both the prevention and treatment of female reproductive disorders. While our results align with previous observational studies to some extent, further validation through larger prospective studies and in-depth mechanistic investigations is necessary to comprehensively elucidate the causal relationship between depression and various types of reproductive conditions.

### Supplementary Information

Supplementary Tables.

Supplementary Figures.

## Data Availability

All data are publicly available. The data sources for this study include the FinnGen consortium (https://www.finngen.fi/fi), the UK Biobank (http://www.nealelab.is/uk-biobank/), and the IEU OpenGWAS database (https://gwas.mrcieu.ac.uk/) .

## Abbreviations

PCOS: Polycystic ovary syndrome
WHO: World Health Organization
RCT: Randomized controlled trial
MR: Mendelian randomization
GWAS: Genome-wide association study
AUB: Abnormal uterine and vaginal bleeding
SNPs: Single nucleotide polymorphisms
IVW: Inverse variance weighting
WM: Weighted median
BMI: Body mass index
HPA: Hypothalamic–pituitary–adrenal
HPO: Hypothalamic–pituitary–ovarian
CRH: Corticotropin-releasing hormone
