# Supplementary material for: Epidemiologic and genetic associations of female reproductive disorders with depression or dysthymia: a Mendelian randomization study
Source: Sci Rep. 2024 Mar 12;14:5984. doi: 10.1038/s41598-024-55993-8 (PMC10933377; doi:10.1038/s41598-024-55993-8)

**Supplementary Information**

**Figure S1.** Scatter plots of significant and nominal significant estimates from genetically predicted depression or dysthymia on (a)ovarian dysfunction; (b) polycystic ovary syndrome; (c) ovarian cysts; (d) abnormal uterine and caginal bleeding ; (e) leiomyoma of the uterus; (f) endometriosis; (g) female infertility; (h) spontaneous abortion; (i) eclampsia; (j) pregnancy hypertension; (k) gestational diabetes; (l) excessive vomiting in pregnancy; (m) cervical cancer; (n) uterine/endometrial cancer.


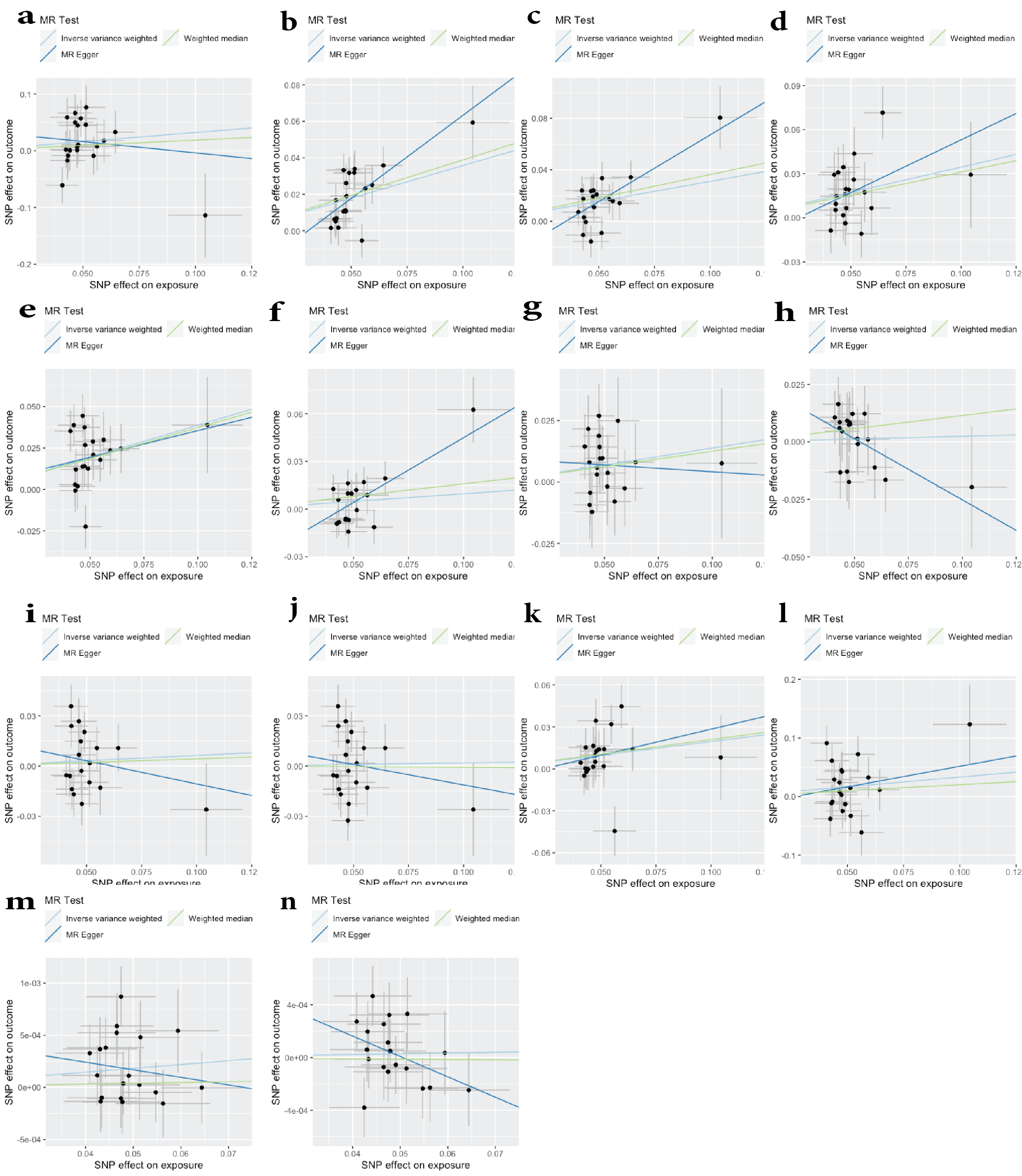


**Figure S2.** Funnel plots of significant and nominal significant estimates from genetically predicted depression or dysthymia on (a)ovarian dysfunction; (b) polycystic ovary syndrome; (c) ovarian cysts; (d) abnormal uterine and caginal bleeding ; (e) leiomyoma of the uterus; (f) endometriosis; (g) female infertility; (h) spontaneous abortion; (i) eclampsia; (j) pregnancy hypertension; (k) gestational diabetes; (l) excessive vomiting in pregnancy; (m) cervical cancer; (n) uterine/endometrial cancer.


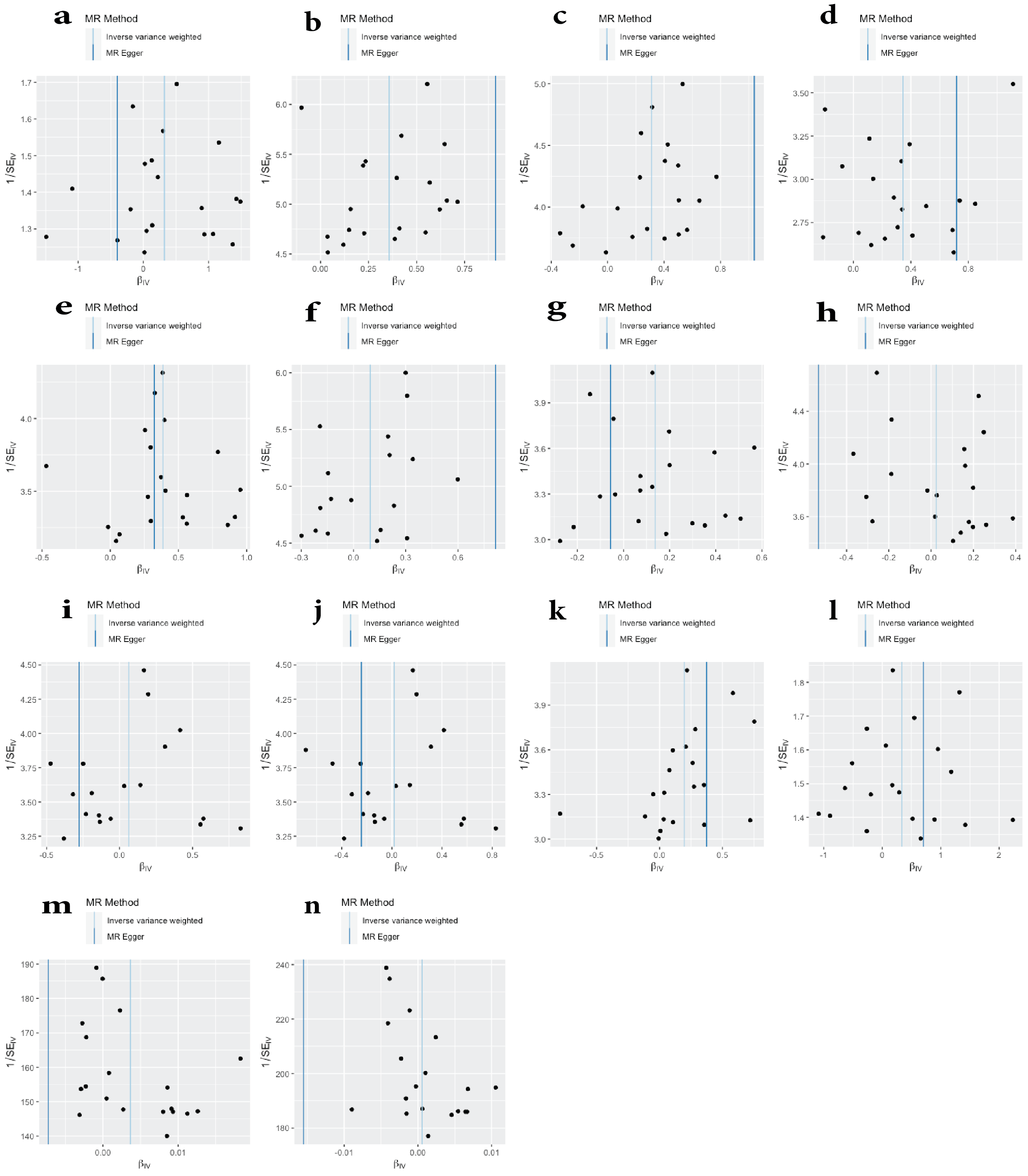


**Figure S3.** Leave-one-out plots of significant and nominal significant estimates from genetically predicted depression or dysthymia on (a)ovarian dysfunction; (b) polycystic ovary syndrome; (c) ovarian cysts; (d) abnormal uterine and caginal bleeding ; (e) leiomyoma of the uterus; (f) endometriosis; (g) female infertility; (h) spontaneous abortion; (i) eclampsia; (j) pregnancy hypertension; (k) gestational diabetes; (l) excessive vomiting in pregnancy; (m) cervical cancer; (n) uterine/endometrial cancer.


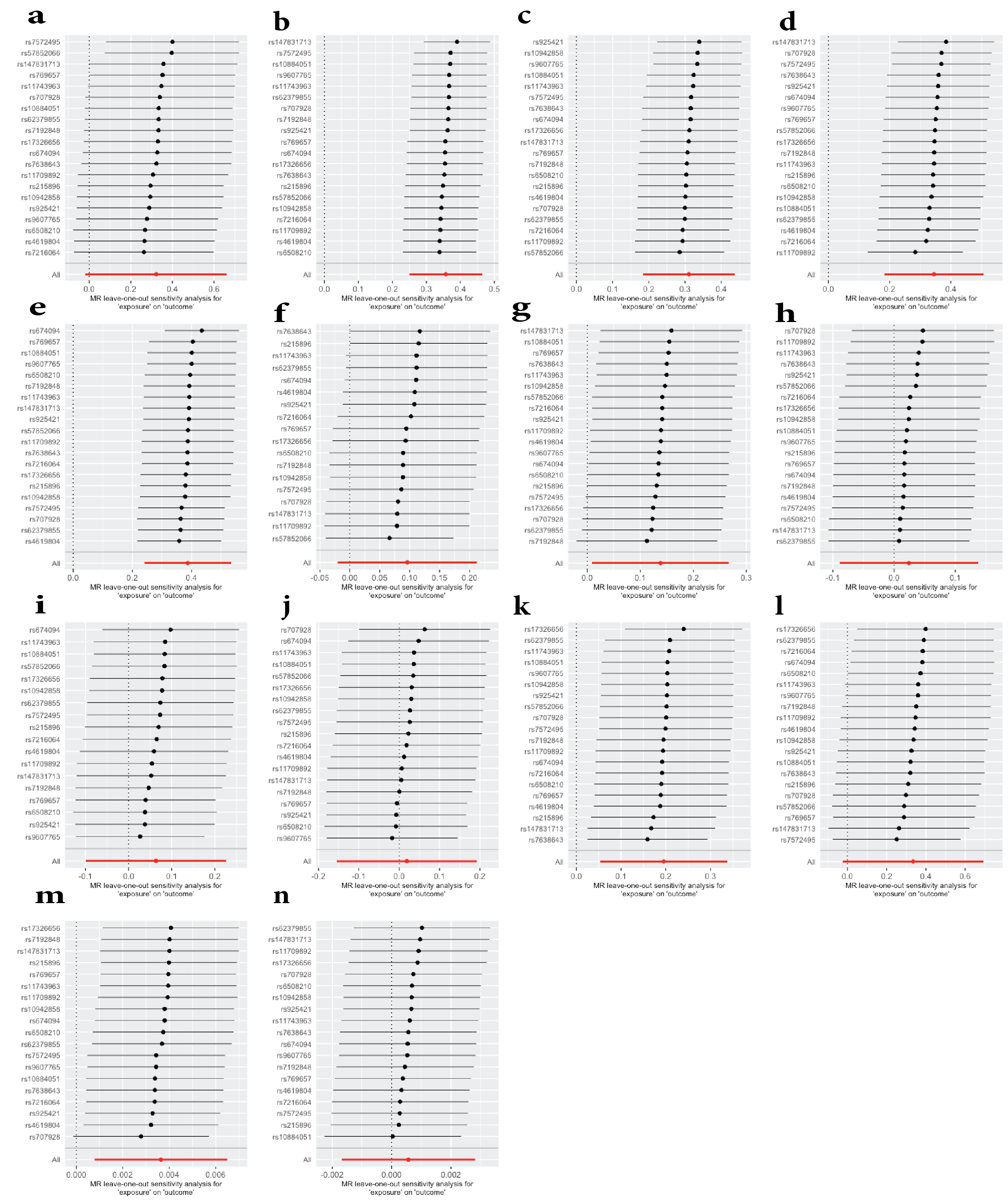


**Figure S4.** Forest plots of significant and nominal significant estimates from genetically predicted depression or dysthymia on (a)ovarian dysfunction; (b) polycystic ovary syndrome; (c) ovarian cysts; (d) abnormal uterine and caginal bleeding ; (e) leiomyoma of the uterus; (f) endometriosis; (g) female infertility; (h) spontaneous abortion; (i) eclampsia; (j) pregnancy hypertension; (k) gestational diabetes; (l) excessive vomiting in pregnancy; (m) cervical cancer; (n) uterine/endometrial cancer.


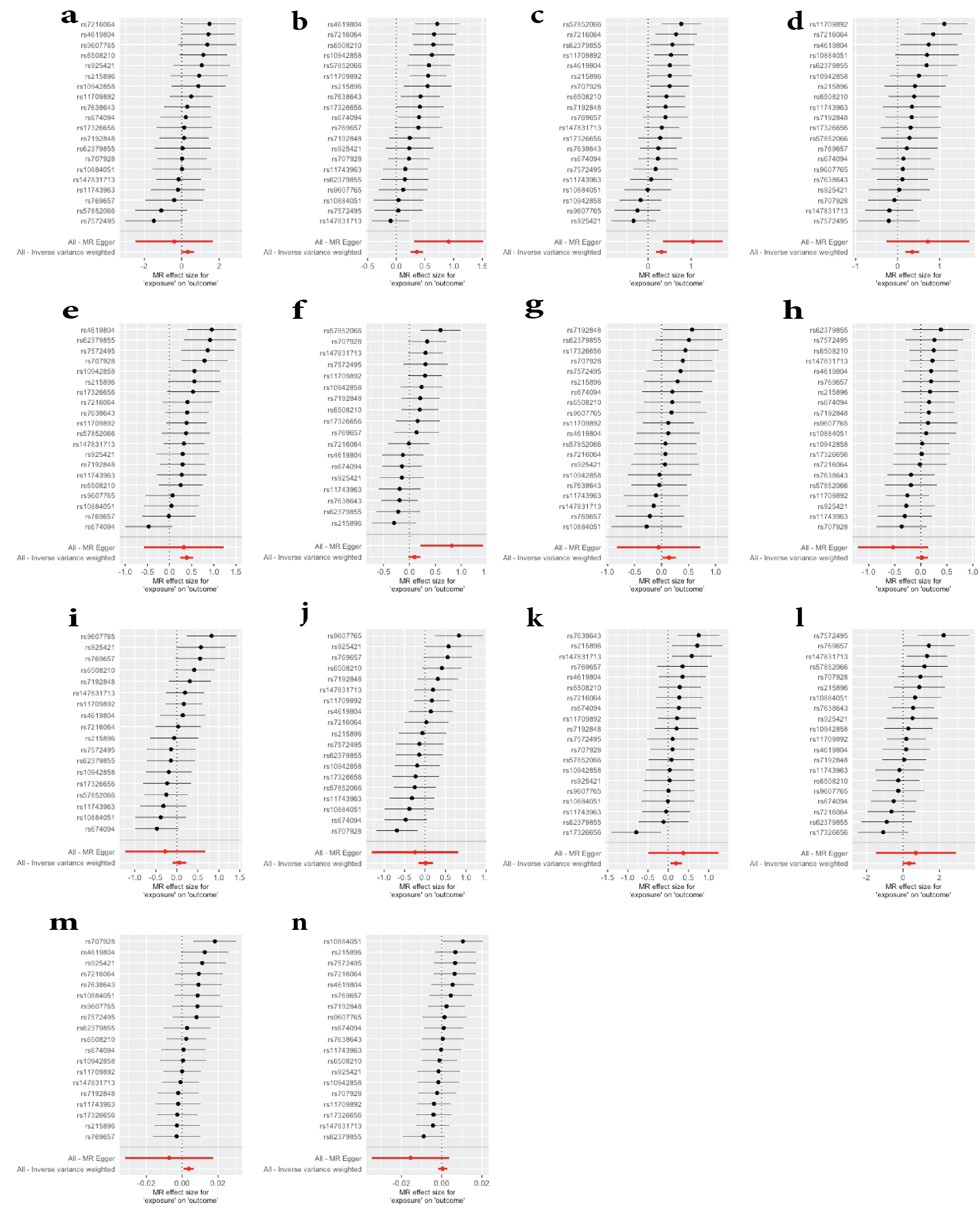

Supplement: Supplementary file 2 — Supplementary Figures. [file 41598_2024_55993_MOESM2_ESM.docx]
